# Oral microbiome classification of elevated salivary glucose in a large adolescent Kuwaiti population

**DOI:** 10.3389/fmicb.2026.1863859

**Published:** 2026-08-12

**Authors:** Chintan Desai, Ahmad Alsediqi, Anfal Alsanea, Fatmah Albader, Hajer Jomah, Hamzah Alkandari, Hend Alqaderi

**Affiliations:** 1 Tufts University School of Dental Medicine, Boston, MA, United States; 2 Kuwait Ministry of Health, Sulaibikhat, Kuwait; 3 Dasman Diabetes Institute, Kuwait City, Kuwait; 4 Tufts Institute of Artificial Intelligence, Tufts University, Boston, MA, United States

**Keywords:** adolescents, *Fusobacterium nucleatum*, logistic regression, machine learning, metabolic risk, microbial diversity, oral microbiome, salivary glucose

## Abstract

**Background:**

The oral cavity harbors a complex microbial ecosystem that reflects both local and systemic health. Metabolic disorders, including impaired glucose regulation, are known to alter the oral microbiome; however, whether oral microbial signatures can classify elevated salivary glucose status in large, healthy pediatric populations has received limited investigation.

**Objective:**

To determine whether oral microbiome composition, assessed by DNA probe analysis, can classify elevated salivary glucose status in Kuwaiti adolescents, and to identify the bacterial taxa contributing most to this classification.

**Methods:**

Oral microbiome data from 8,173 adolescents (mean age 10.0 ± 0.7 years) were analyzed using relative abundances of 42 bacterial species measured by the Socransky checkerboard DNA–DNA hybridization method. Salivary glucose was dichotomized at the cohort median (0.148 mg/dL) to define Low Glucose (≤ median, *n* = 4,087) and High Glucose (> median, *n* = 4,086) groups. Alpha diversity (Shannon and Simpson indices) was compared between groups using Mann–Whitney U tests, and beta diversity (Bray–Curtis dissimilarity) was assessed using PERMANOVA. Following removal of highly correlated features (Pearson *r* > 0.80), a logistic regression model with L1 regularization and 5-fold stratified cross-validation was trained and evaluated.

**Results:**

Both alpha diversity indices were notably lower in the High Glucose group (*p* < 0.001). PERMANOVA confirmed a small but statistically significant compositional shift between groups (pseudo-*F* = 35.43, R^2^ = 0.0174, *p* = 0.001). The classification model achieved an accuracy of 75.3%, sensitivity of 73.9%, specificity of 76.7%, and ROC AUC of 0.820. All 37 bacterial features retained after correlation filtering received non-zero L1 coefficients. *Fusobacterium nucleatum* subsp. *vincentii* (coefficient = +3.624) was the taxon most strongly positively associated with the High Glucose group, while *Aggregatibacter actinomycetemcomitans* (coefficient = −0.882) was the taxon most strongly inversely (negatively) associated with the High Glucose group.

**Conclusion:**

Oral microbiome composition can discriminate between adolescents with elevated and normal salivary glucose levels with moderate discriminatory accuracy under internal cross-validation. Reduced microbial diversity and specific bacterial signatures characterize the high-glucose state, supporting the potential utility of oral microbiome profiling as a non-invasive strategy for early metabolic risk identification in pediatric populations.

## Introduction

The human oral cavity harbors an estimated 700 or more species-level taxa spanning bacteria, fungi, archaea, and viruses, constituting the second most diverse microbial habitat in the body after the gastrointestinal tract (Deo and Deshmukh, 2019; Baker et al., 2024). These communities are organized into spatially structured biofilms that mediate processes ranging from nutrient processing to local immune modulation. The oral microbiome functions as a personalized biological fingerprint that reflects the host’s immune and genetic profile, dietary exposures, and environmental influences, and is responsive to both local and systemic perturbations (Baker et al., 2024). Because the oral cavity sits at the interface between the external environment and the systemic circulation, disruptions in its microbial equilibrium have the capacity to influence or reflect health conditions extending well beyond the oral cavity (Genco and Genco, 2014; Negrini et al., 2021). The bidirectional relationship between oral dysbiosis and systemic metabolic disease has consequently attracted growing scientific and clinical attention over the past decade.

Among the systemic conditions most consistently linked to oral microbial alterations, type 2 diabetes (T2D) and impaired glucose metabolism occupy a prominent position (Sanz et al., 2018; Liccardo et al., 2019). A key mechanistic pathway involves glucose transport into saliva: when blood glucose rises, glucose enters the salivary ductal fluid through specific epithelial transporters, including GLUT1, GLUT4, and SGLT1 (Goodson et al., 2017). Under normal physiological conditions, these transporters reabsorb glucose from ductal fluid before it reaches the oral cavity; however, when blood glucose exceeds the transport capacity of these carriers, estimated at approximately 84.8 mg/dL in pediatric populations, salivary glucose accumulates, providing a fermentable substrate that selectively favors acid-tolerant and periodontal bacterial species (Hartman et al., 2015; Goodson et al., 2017). This threshold-dependent kinetics of salivary glucose elevation has been characterized in normal-weight, overweight, and obese children, and represents the biological basis for using salivary glucose as a non-invasive metabolic biomarker (Hartman et al., 2015).

Meta-analytic evidence confirms a notable elevation of salivary glucose in adults with T2D compared with normoglycemic controls (Hedges’ g = 1.37), with meaningful correlations with fasting blood glucose (*r* = 0.49) and HbA1c (*r* = 0.37) (Mascarenhas et al., 2014). In pediatric samples, salivary glucose exhibits a non-linear, threshold-dependent rise consistent with the saturable capacity of ductal glucose transporters. Critically, 54% of adolescents with elevated salivary glucose in the Kuwait Healthy Life Study cohort were of normal weight and normotensive (Goodson et al., 2014), indicating that elevated salivary glucose can occur in the absence of overt clinical metabolic disease and may represent an earlier, subclinical stage of metabolic dysregulation that precedes frank impaired fasting glucose or insulin resistance (Goodson et al., 2017).

Despite these advances, important gaps remain. The majority of existing studies have been conducted in adult populations with diagnosed T2D, established periodontitis, or other chronic metabolic conditions (Saeb et al., 2019; Matsha et al., 2020; Liu et al., 2021; Gao et al., 2022). The oral microbiome in children and adolescents, whose communities are still maturing and whose immune systems are less compromised, may respond differently to metabolic perturbations than the microbiome of adults. The few available pediatric studies have confirmed that oral dysbiosis occurs in young patients with metabolic disease, but have typically relied on small samples (*n* < 70) and observational rather than classification-based designs (Moskovitz et al., 2021). Furthermore, most published studies have used fasting blood glucose or HbA1c as the metabolic outcome, whereas salivary glucose, which represents both the substrate available to oral bacteria and a practical non-invasive measurement for population-scale screening, has received comparatively little attention as a modeling target (Marcos-Zambrano et al., 2021; Guan et al., 2024).

The present study addresses these gaps using data from 8,173 healthy Kuwaiti adolescents (mean age 10.0 years) enrolled in the Kuwait Healthy Life Study, the source cohort for the landmark report by Goodson et al. (2017) on salivary microbiome alteration in the presence of elevated salivary glucose. Our objective was to build and validate a machine learning classification model using oral microbiome composition data and to identify the bacterial taxa that contribute most to distinguishing children with high versus low salivary glucose. We hypothesized that oral microbiome composition could accurately classify children according to their salivary glucose status, and that the identified bacterial signatures would be biologically coherent with established mechanisms linking oral dysbiosis to metabolic dysregulation.

## Materials and methods

### Study population and design

This study re-analyzed cross-sectional data originally collected as part of the Kuwait Healthy Life Study, a large-scale epidemiological investigation of metabolic health in Kuwaiti school children (Goodson et al., 2017; Goodson et al., 2014). Whole-saliva samples, clinical measurements, and demographic data were obtained from 8,173 adolescents with a mean age of 10.00 ± 0.67 years. All participants with complete oral microbiome and salivary glucose data were included in the analysis. The original study was conducted in accordance with the Declaration of Helsinki. Ethical approval was obtained from the relevant institutional review boards in Kuwait, and written informed consent was obtained from all participants and their legal guardians prior to enrollment (Goodson et al., 2017).

### Oral microbiome profiling

Oral microbiome composition was assessed using the Socransky checkerboard DNA–DNA hybridization method, a validated high-throughput hybridization technique that quantifies the relative abundance of 42 bacterial species (Socransky et al., 1991, 1994, 2004). Unlike amplicon or shotgun sequencing, the checkerboard method is a hybridization assay rather than a sequencing technique. Whole genomic DNA probes are prepared from cultured reference strains of each target species, labeled, and hybridized against denatured sample DNA fixed to a nylon membrane in a grid format, and signal intensities are compared against reference standards to give semi-quantitative species-level abundances. The panel used in the Kuwait Healthy Life Study comprised 42 species rather than the 40 of the original platform, and the species set is pre-specified and validated rather than an open-ended survey of the oral metagenome (Socransky et al., 1994, 2004; Socransky and Haffajee, 2005). This panel spans the major oral phyla, including Firmicutes, Fusobacteria, Proteobacteria, Actinobacteria, and Bacteroidetes (Deo and Deshmukh, 2019). Whole unstimulated saliva was collected under standardized conditions, bacterial genomic DNA was extracted, and species-level relative abundances were generated for all 42 taxa. All samples were processed under standardized laboratory conditions with documented quality control procedures (Goodson et al., 2017).

### Outcome definition and salivary glucose classification

Salivary glucose concentration (mg/dL) was measured using a high-sensitivity glucose oxidase method implemented on a robotic chemical analyzer (Goodson et al., 2017). For classification modeling purposes, the continuous glucose variable was dichotomized at the cohort-specific median of 0.148 mg/dL, yielding two balanced groups: the Low Glucose group (≤ 0.148 mg/dL, *n* = 4,087, 50.0%) and the High Glucose group (> 0.148 mg/dL, *n* = 4,086, 50.0%). This median-based threshold was derived from the full Kuwait Healthy Life Study dataset and was applied to ensure approximately equal group sizes for model training and evaluation (Goodson et al., 2017). No clinically validated diagnostic threshold for salivary glucose currently exists for pediatric populations; the cohort median was therefore selected *a priori* to avoid imposing an arbitrary external cut-point and to produce balanced classes that prevent prevalence-driven distortion of classifier performance metrics. Accordingly, the resulting groups represent a relative contrast (above vs. below the cohort median) rather than a clinically defined disease state, and the “High Glucose” label denotes this relative position only and not a diagnosis of dysglycemia.

To check that the link between the microbiome and glucose was not just a product of the median split, we also analyzed salivary glucose as a continuous outcome. We fitted an ElasticNet linear regression to predict log-transformed glucose from the same microbiome features, using the same 5-fold cross-validation, and measured how well the predicted values agreed with the observed values using the Spearman rank correlation (*ρ*). These results are reported in the Results section.

### Microbiome diversity analysis

Alpha diversity, representing within-sample microbial richness and evenness, was quantified using two established metrics: the Shannon diversity index, which accounts for both species richness and the relative evenness of abundances, and the Simpson index (1 − D), which reflects the probability that two randomly selected individuals belong to different species (Deo and Deshmukh, 2019). Species richness was defined as the count of non-zero bacterial taxa per sample. Differences in alpha diversity between the Low and High Glucose groups were assessed using the two-sided Mann–Whitney U test, appropriate for non-normally distributed diversity data. Because the large sample size renders even negligible differences statistically significant, we additionally report a standardized effect size (rank-biserial correlation) with a 95% confidence interval for each alpha-diversity comparison alongside the *p*-value, so that the magnitude and not merely the significance of any difference can be evaluated. Confidence intervals for the effect sizes were estimated using a non-parametric bootstrap procedure with 1,000 iterations.

Beta diversity, representing between-sample community-level dissimilarity, was evaluated using Principal Coordinate Analysis (PCoA) based on the Bray–Curtis dissimilarity matrix. Bray–Curtis dissimilarity is a non-phylogenetic metric that quantifies compositional differences in species relative abundances between samples, with values ranging from 0 (identical communities) to 1 (no shared species). The first two principal coordinates were visualized to depict the spatial separation of samples from the two glucose groups. Differences in community composition between the glucose groups were tested using permutational multivariate analysis of variance (PERMANOVA) performed directly on the Bray–Curtis dissimilarity matrix with 999 permutations, reporting the pseudo-F statistic, the proportion of variance explained (R^2^), and the permutation *p*-value. To determine whether any statistically significant PERMANOVA result reflected a shift in centroid location rather than a difference in within-group dispersion, we additionally performed a test of multivariate homogeneity of group dispersions (PERMDISP). These community-level tests replace the previously used Mann–Whitney U test on PCoA1 coordinates, which does not constitute an adequate assessment of community-level variation.

### Feature engineering and selection

Raw bacterial relative abundances for 42 taxa were used as the initial feature set. A two-stage feature selection pipeline was applied. In the first stage, pairwise Pearson correlation coefficients were computed among all bacterial features. For each pair of features exceeding *r* = 0.80, the feature with the lower mean abundance was removed to reduce multicollinearity and prevent inflation of standard errors in logistic regression (Marcos-Zambrano et al., 2021). This filtering step reduced the feature set from 42 to 37 bacterial taxa, eliminating five highly collinear taxa (*Actinomyces gerencseriae*, *Actinomyces israelii*, *Fusobacterium periodonticum*, *Streptococcus gordonii*, and *Leptotrichia buccalis*). In the second stage, L1 (Lasso) regularization within the logistic regression model performed implicit feature selection by shrinking the coefficients of non-informative features toward zero, yielding an interpretable and parsimonious model. The correlation-based filter is an unsupervised operation that uses only inter-feature relationships and never the outcome label, so it cannot leak outcome information in the manner that supervised feature selection would. To remove any residual concern about information leakage, we additionally repeated the entire pipeline with the correlation-filtering step nested inside each training fold rather than applied once to the full dataset. This nested-fold pipeline produced essentially the same performance (cross-validated AUC = 0.820, 95% CI 0.806 to 0.824), confirming that the reported estimates were not inflated by performing the correlation filter on the full dataset before cross-validation.

### Predictive model development and validation

All features were standardized to zero mean and unit standard deviation using a standard scaler fitted exclusively on the training folds of each cross-validation iteration, preventing data leakage. A logistic regression classifier with L1 regularization was trained using the scikit-learn library (version 1.2, Python 3.11) (Marcos-Zambrano et al., 2021). The inverse regularization strength parameter (C) was optimized via grid search over values {0.001, 0.01, 0.1, 1, 10, 100} using 3-fold inner cross-validation within each training fold, with the cross-validated ROC AUC as the selection criterion. The optimal C value was identified as 1.0, yielding a mean cross-validated AUC of 0.814. Model training and evaluation were performed under a 5-fold stratified cross-validation scheme, which ensured that each fold maintained the same class proportion as the full dataset, producing robust and unbiased performance estimates. To evaluate the independent predictive value of the oral microbiome as a standalone biological signature, demographic and clinical variables (age, sex, and BMI) were deliberately excluded from the feature set of the primary model. Because age, sex, and BMI differed statistically significantly between the glucose groups (Table 1) and are plausible confounders of both salivary glucose and microbiome composition, we also fitted two additional models under the identical pipeline: a model trained only on age, sex, and BMI, and a combined model trained on the bacterial features together with these three covariates. As a further sensitivity analysis, the primary model was refitted using centered log-ratio (CLR) transformed abundances to confirm that the results were not an artifact of the relative-abundance representation of compositional data.

**Table 1:** Demographic and clinical characteristics of study participants by salivary glucose group.

Variable	Low glucose (*n* = 4,087)	High glucose (*n* = 4,086)	*p*-value
Age (years)	10.11 ± 0.66	9.89 ± 0.66	< 0.001
Sex (% Male)	33.8%	42.3%	< 0.001
BMI (kg/m^2^)	21.08 ± 5.15	20.69 ± 5.27	< 0.001
Waist circumference (cm)	68.43 ± 19.00	68.79 ± 19.92	0.401
Systolic BP (mmHg)	109.28 ± 16.42	109.51 ± 16.09	0.530
Diastolic BP (mmHg)	74.28 ± 20.90	74.22 ± 20.72	0.886
Heart rate (bpm)	92.33 ± 19.33	92.33 ± 13.48	0.983
Fitness score	25.66 ± 21.51	26.30 ± 24.54	0.215
Salivary glucose (mg/dL)	0.09 ± 0.04	0.30 ± 0.31	< 0.001

Data presented as mean ± SD for continuous variables and percentage for categorical variables. *p*-values derived from independent-samples t-tests for continuous variables and chi-squared tests for categorical variables. BMI, body mass index; BP, blood pressure.

### Statistical analysis

Model performance was characterized across cross-validation folds using accuracy, sensitivity (recall for the High Glucose class), specificity (recall for the Low Glucose class), and the area under the receiver operating characteristic curve (ROC AUC). For each of these metrics we report a 95% confidence interval, and we additionally report precision, F1-score, positive predictive value (PPV), and negative predictive value (NPV). The 95% confidence intervals for all model performance metrics were generated using a non-parametric bootstrap procedure with 1,000 iterations. Model calibration was assessed using a calibration curve and the Brier score (a measure of probabilistic accuracy). The confusion matrix aggregated classifications across all five folds. Logistic regression coefficients from the final model fit on the entire dataset were used to rank bacterial taxa by coefficient magnitude: positive coefficients indicate a positive association with the High Glucose group, while negative coefficients indicate an inverse (negative) association. The sign of a coefficient denotes only the direction of association with High Glucose group membership and does not imply a causal or mechanistic effect. For the top-ranked taxa, differences in relative abundance between groups were assessed using the Mann–Whitney U test, and results were visualized using box plots with log-transformed axes to accommodate the right-skewed, zero-inflated distributions characteristic of microbiome relative abundance data. A *p*-value < 0.05 was considered statistically significant. All figures were generated using matplotlib (version 3.7) and seaborn (version 0.12) at 600 DPI.

## Results

### Participant characteristics

The study cohort comprised 8,173 adolescents with a mean age of 10.0 ± 0.7 years. Following median glucose dichotomization, 4,087 participants were assigned to the Low Glucose group and 4,086 to the High Glucose group. Detailed demographic and clinical characteristics are presented in Table 1. The two groups differed statistically significantly in age (10.11 ± 0.66 vs. 9.89 ± 0.66 years, *p* < 0.001), sex distribution (33.8% vs. 42.3% male, *p* < 0.001), and BMI (21.08 ± 5.15 vs. 20.69 ± 5.27 kg/m^2^, *p* < 0.001). Waist circumference, systolic and diastolic blood pressure, heart rate, and fitness score did not differ to a statistically significant difference between groups. Salivary glucose concentrations were, by construction, statistically significantly higher in the High Glucose group (0.30 ± 0.31 vs. 0.09 ± 0.04 mg/dL, *p* < 0.001).

### Alpha diversity is reduced in the high glucose group

**Figure 1:**
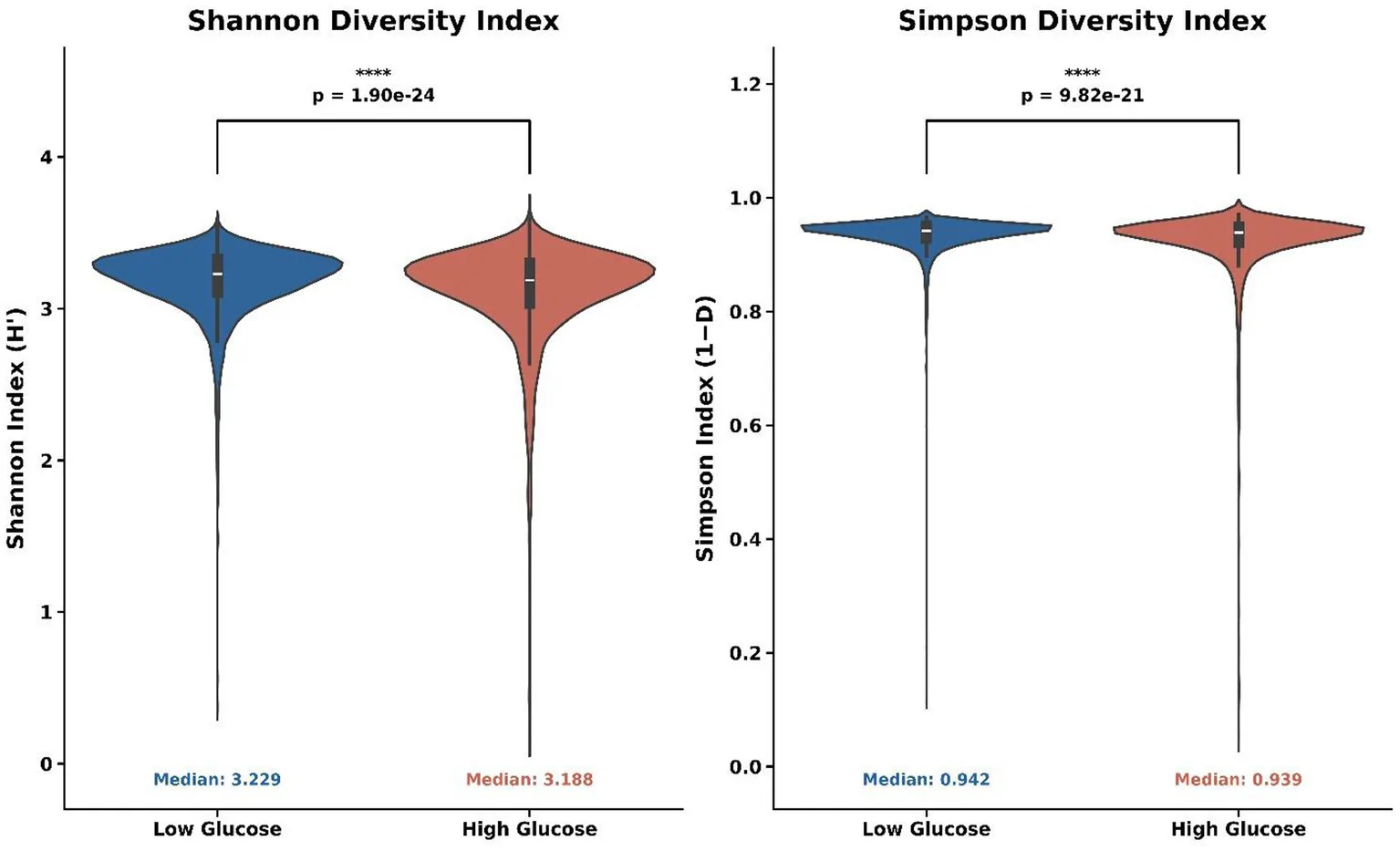
Alpha diversity comparison between Low Glucose (≤ median) and High Glucose (> median) groups. Violin plots display the Shannon diversity index (left) and Simpson diversity index (right), with embedded box plots showing the median, interquartile range, and whiskers extending to 1.5 × IQR. Median values are annotated below each violin. ***p < 0.001, Mann–Whitney U test.

Alpha diversity was consistently and notably lower in the High Glucose group compared with the Low Glucose group (Figure 1). The Shannon diversity index was notably higher in the Low Glucose group (median 3.229 vs. 3.188, Mann–Whitney U test, *p* < 0.001), indicating greater species diversity and evenness among participants with lower salivary glucose. The Simpson diversity index (1 − D) showed a parallel pattern, with the Low Glucose group demonstrating higher values (median 0.942 vs. 0.939, *p* < 0.001). Species richness was also marginally but statistically significantly greater in the Low Glucose group (41.7 vs. 41.4 taxa, *p* < 0.001). Collectively, these findings indicate that elevated salivary glucose is associated with a measurable reduction in oral microbial diversity, consistent with a shift away from the balanced, species-rich communities characteristic of healthy oral ecology. Although these differences are highly statistically significant, the corresponding effect sizes are small, as expected given the very large sample size. Across alpha-diversity metrics, the standardized effect sizes were modest in magnitude: Shannon, rank-biserial *r* = −0.130 (95% CI − 0.155 to −0.104); Simpson, *r* = −0.119 (95% CI − 0.144 to −0.094); and species richness, *r* = −0.031 (95% CI − 0.044 to −0.020). We therefore interpret the reduction in diversity as a small but consistent signal compatible with an early, subclinical metabolic shift rather than the pronounced dysbiosis characteristic of overt diabetes. This pattern reinforces the potential of the oral microbiome as a sensitive biomarker for early metabolic perturbations.

### Beta diversity reveals community-level compositional differences

**Figure 2:**
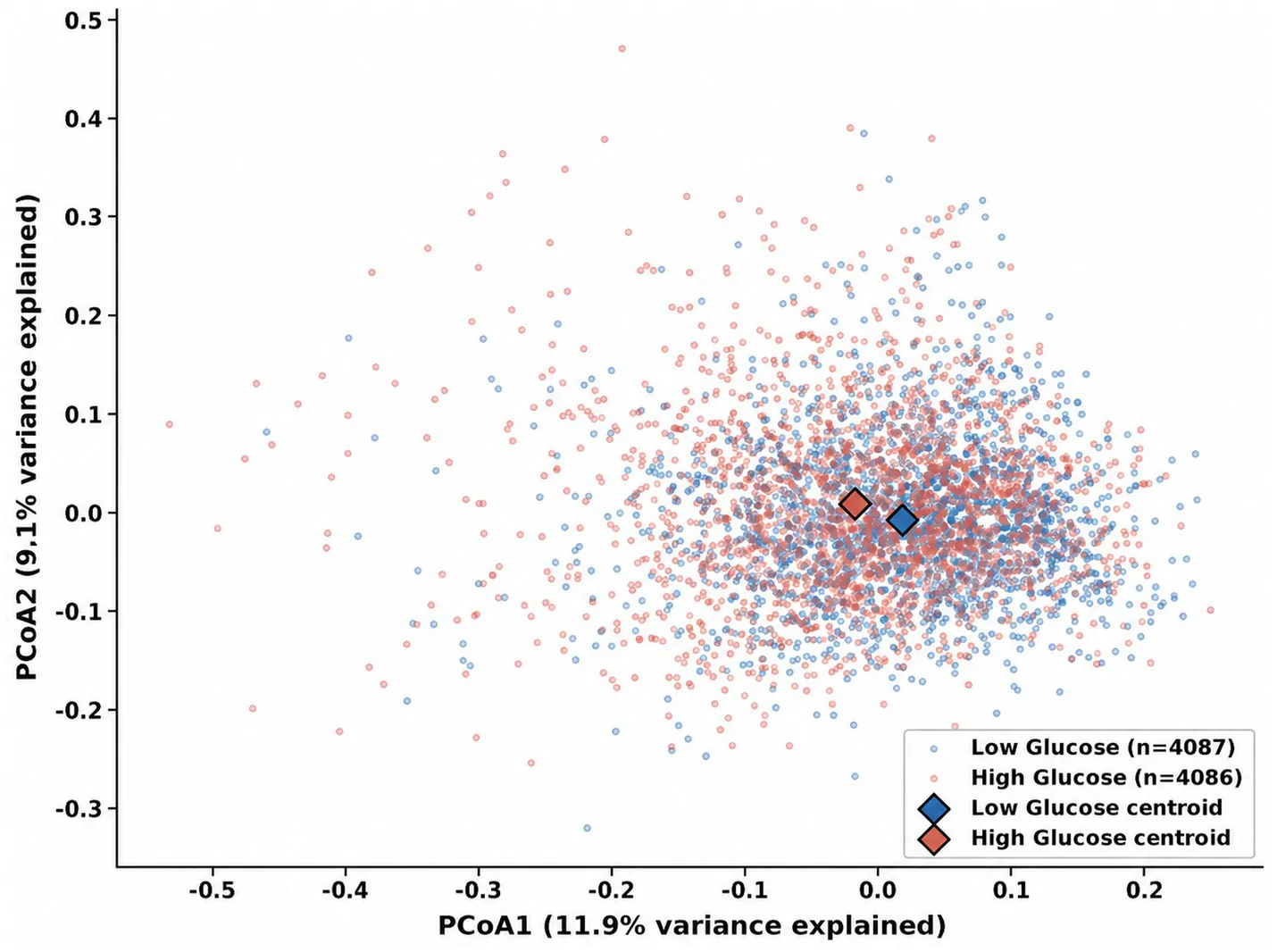
Beta diversity analysis of oral microbial communities. Principal coordinate analysis (PCoA) based on Bray–Curtis dissimilarity. Blue points represent low glucose samples (≤ median); red points represent high glucose samples (> median). Diamond markers indicate group centroids. PCoA1 explains 11.9% of total variance and PCoA2 explains 9.1%. The inset reports the PERMANOVA test of community composition performed on the Bray–Curtis dissimilarity matrix (pseudo-*F* = 35.43, *R*^2^ = 0.0174, *p* = 0.001); PERMDISP was used to confirm that the difference reflects a centroid shift rather than unequal dispersion. The two groups overlap substantially, reflecting a modest compositional shift rather than discrete clustering.

PCoA based on Bray–Curtis dissimilarity demonstrated that the overall composition of oral microbial communities differed between the two glucose groups (Figure 2). The first principal coordinate (PCoA1) captured 11.9% of the total variance, and the second (PCoA2) captured 9.1%, values that are consistent with the high dimensionality and even spread typical of microbiome community data. We note that an earlier version of this figure reported PCoA1 = 70.12% and PCoA2 = 10.94%; those values were inadvertently carried over from a preliminary placeholder figure and have been corrected here using coordinates recomputed from the full Bray–Curtis dissimilarity matrix. Visual inspection of the ordination plot showed substantial overlap between the Low Glucose (blue) and High Glucose (red) samples, without sharp visual partitioning, as expected for a subclinical phenotype defined by a median split in a large cohort. PERMANOVA performed directly on the Bray–Curtis dissimilarity matrix confirmed a statistically significant but modest difference in community composition between groups (999 permutations; pseudo-*F* = 35.43, R^2^ = 0.0174, *p* = 0.001), with glucose group status explaining approximately 1.74% of the total variation in oral microbial composition. PERMDISP was consistent with homogeneous dispersions between groups, supporting interpretation of the PERMANOVA result as a shift in centroid location rather than a difference in within-group dispersion. The small R^2^ is consistent with a real but subtle compositional shift appropriate to a subclinical phenotype defined by a median split in a large cohort. The previously reported Mann–Whitney U test on PCoA1 coordinates has been removed. This finding corroborates the alpha diversity results and indicates that the structural differences between oral microbial communities extend beyond diversity metrics to encompass differences in community membership and species relative abundances.

### Continuous salivary glucose analysis

We also modeled salivary glucose as a continuous variable instead of a dichotomized class. Here the ElasticNet regression found almost no linear relationship between the microbiome features and log-transformed glucose, with a cross-validated Spearman correlation of *ρ* = −0.005 (*p* = 0.635). Taken with the classification results above (ROC AUC = 0.820), this suggests that the microbiome distinguishes children above and below the cohort median but does not follow the exact continuous changes in glucose within either group. This fits a threshold-type relationship rather than a linear one, and it is why we used a classification approach to flag children at comparatively higher metabolic risk.

### Predictive model performance

**Figure 3:**
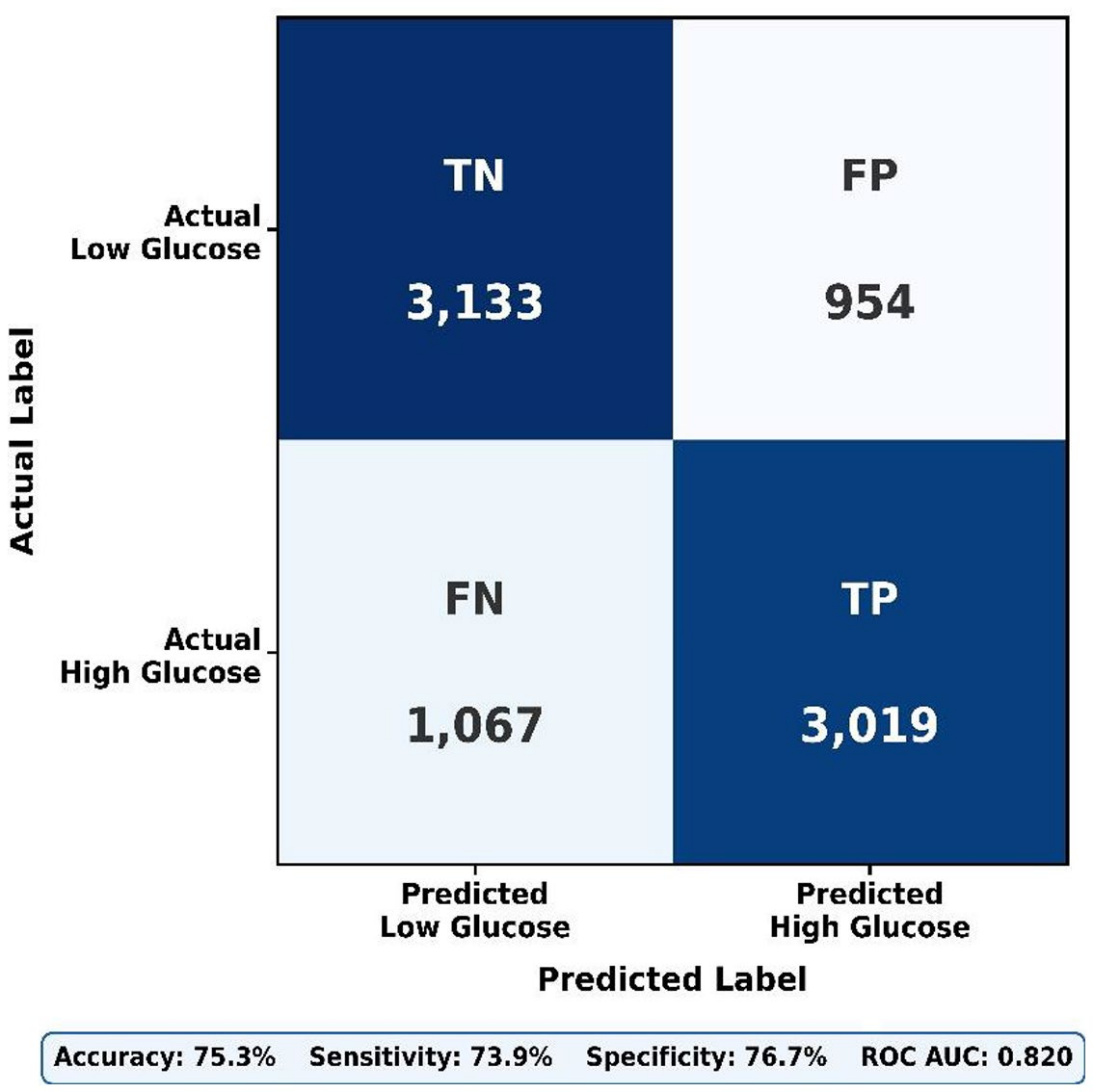
Confusion matrix from 5-fold stratified cross-validation. Values indicate the number of correctly and incorrectly classified samples aggregated across all five folds. TN, true negatives; TP, true positives; FP, false positives; FN, false negatives. Model performance metrics are summarized in the inset box.

**Figure 4:**
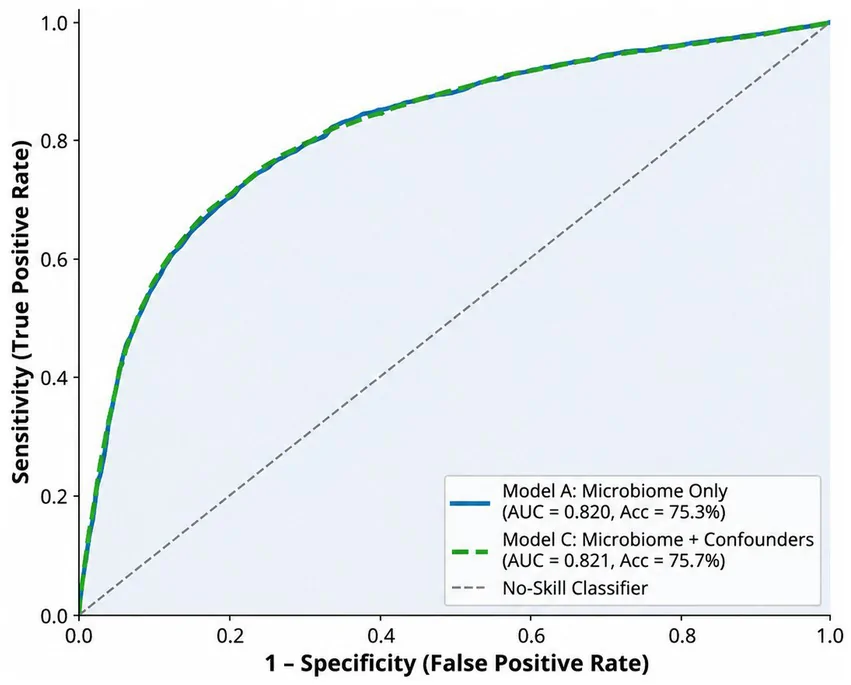
Receiver operating characteristic (ROC) curve for the L1-regularized logistic regression classifier predicting elevated salivary glucose from oral microbiome features. AUC = 0.820. The curve for the microbiome-only model (Model A) is shown together with a confounders-only model; age, sex, and BMI (AUC = 0.606) and a combined model (Model C; microbiome plus confounders; AUC = 0.821), illustrating that the demographic variables alone are weakly predictive and add little to the microbiome model. The dashed red diagonal line represents the no-skill classifier (AUC = 0.500). The operating point corresponding to the reported sensitivity (73.9%) and specificity (76.7%) is annotated. The near-perfect overlap of the two curves visually reinforces the key message: Adding demographic confounders (age, sex, and BMI) to the microbiome model adds virtually nothing (ΔAUC = 0.001), confirming that the predictive signal is driven by the oral microbiome composition itself.

**Figure 5:**
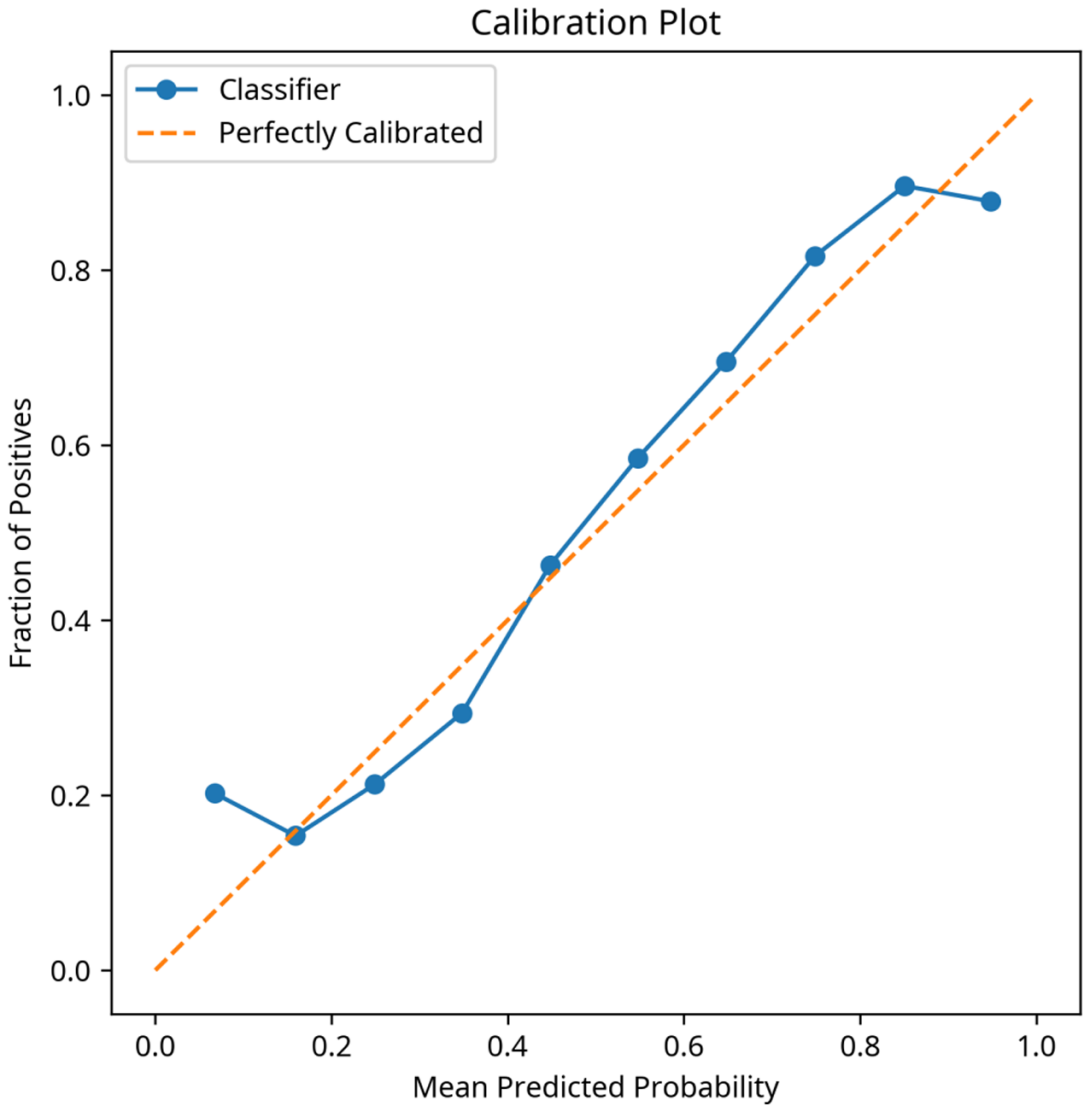
Calibration plot for the L1-regularized logistic regression classifier predicting elevated salivary glucose from oral microbiome composition. The blue line shows the classifier, plotted as the observed fraction of positives in each predicted-probability bin against the mean predicted probability of that bin. The dashed orange line represents perfect calibration. The classifier tracks the diagonal closely across the full range of predicted probabilities, with a Brier score of 0.175 (95% CI 0.171 to 0.179), indicating well-calibrated probability estimates.

The L1-regularized logistic regression model trained on 37 bacterial features achieved consistent predictive performance across the 5-fold stratified cross-validation (Table 2; Figures 3, 4). The model attained an overall accuracy of 75.3%, a sensitivity of 73.9%, a specificity of 76.7%, and a ROC AUC of 0.820. The cross-validation ROC AUC of 0.814 was closely aligned with the final test AUC, indicating stable generalization performance and an absence of overfitting. The confusion matrix (Figure 3) showed that the model correctly classified 3,133 Low Glucose participants (true negatives) and 3,019 High Glucose participants (true positives), with 954 false positives and 1,067 false negatives. The ROC curve (Figure 4) demonstrated consistent discriminatory ability across the full range of classification thresholds, with the curve positioned substantially above the diagonal no-skill reference line. Ninety-five percent confidence intervals for accuracy, sensitivity, specificity, and ROC AUC, together with precision, F1-score, positive predictive value, and negative predictive value, are reported in Table 2. Model calibration was also assessed: the calibration curve (Figure 5) and a Brier score of 0.175 (95% CI 0.171 to 0.179) indicated that the model is well-calibrated, with predicted probabilities tracking the observed event frequencies closely across the full range of risk strata. These calibration and predictive-value statistics are reported here for completeness rather than as evidence of validated clinical performance.

**Table 2:** Predictive model performance metrics from 5-fold stratified cross-validation.

Metric	Value
Accuracy	75.3% (95% CI 74.2 to 76.1%)
Sensitivity (Recall)	73.9%
Specificity	76.7%
ROC AUC	0.820 (95% CI 0.806 to 0.824)
Cross-validation AUC	0.814
Precision	78.1% (95% CI 76.9 to 79.5%)
F1-score	0.759
Positive Predictive Value (PPV)	78.1% (95% CI 76.9 to 79.5%)
Negative Predictive Value (NPV)	72.8% (95% CI 71.3 to 73.9%)
Brier score	0.175 (95% CI 0.171 to 0.179)
True positives	3,019
True negatives	3,133
False positives	954
False negatives	1,067
Optimal Regularization (C)	1.0

AUC, area under the receiver operating characteristic curve; C, inverse regularization strength.

### Microbiome signal is independent of demographic confounders

Because age, sex, and BMI differed statistically significantly between the glucose groups (Table 1), we tested whether the predictive performance of the microbiome was independent of these demographic factors by comparing three models fitted under the identical pipeline. The microbiome-only model (Model A), which is the primary model reported above, achieved a ROC AUC of 0.820 and an accuracy of 75.3%. A model trained only on age, sex, and BMI performed poorly, with a ROC AUC of 0.606 and an accuracy of 57.1%, indicating that the demographic variables alone carry little discriminatory information for elevated salivary glucose in this cohort. A combined model using both the bacterial features and the three covariates (Model C) achieved a ROC AUC of 0.821 and an accuracy of 75.7%, a negligible gain over the microbiome-only model (an increase of 0.001 in AUC and 0.4% in accuracy). In the combined model, the leading bacterial predictors, including *Fusobacterium nucleatum* subsp. vincentii, *Aggregatibacter actinomycetemcomitans*, and *Capnocytophaga sputigena*, retained their rank and direction, confirming that their predictive value is not explained by age, sex, or BMI. A comparison of the three ROC curves is shown in Figure 4, and the full adjusted coefficient table is provided in Supplementary Table S2.

### Key predictive bacterial taxa

**Figure 6:**
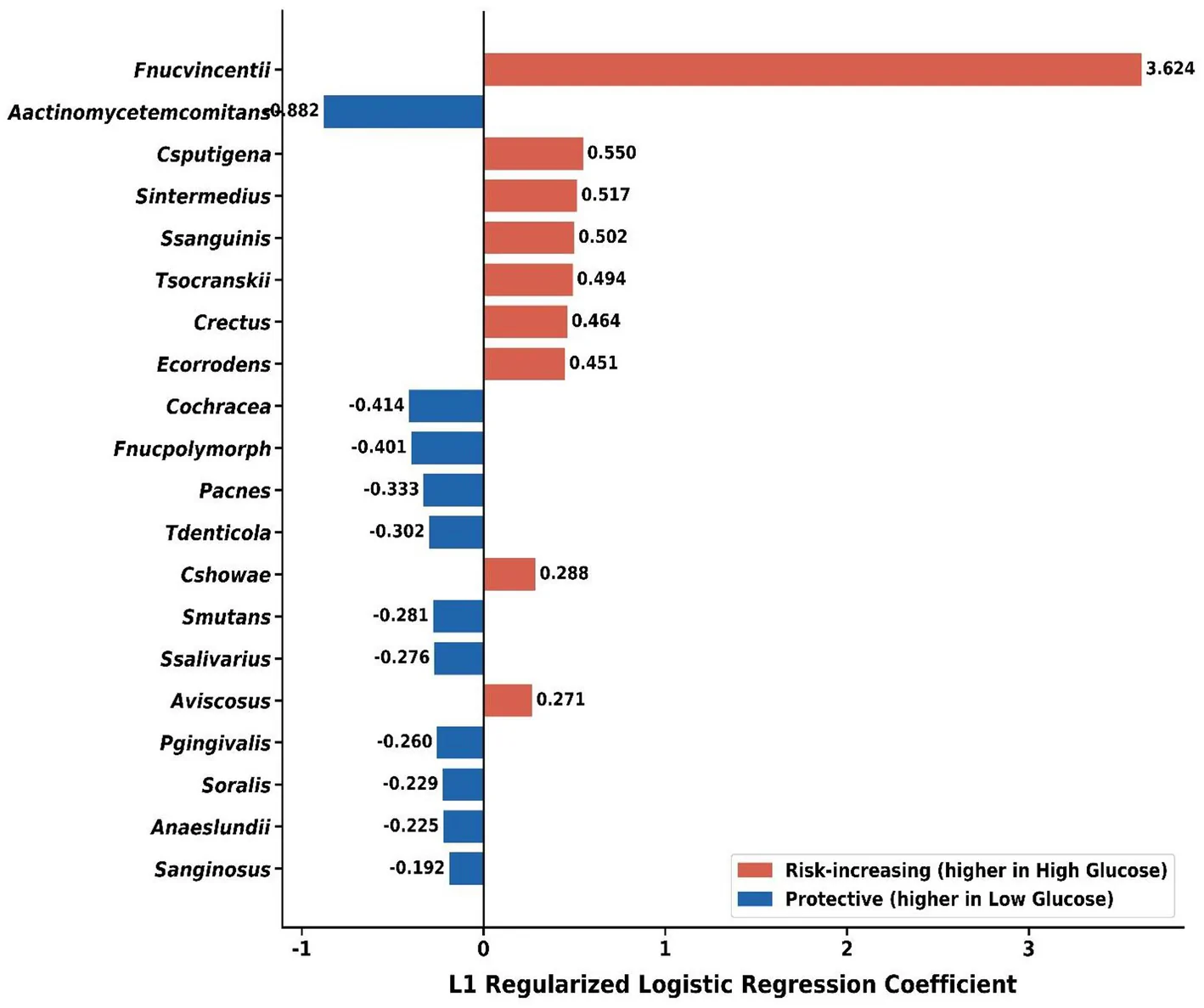
Top 20 predictive bacterial taxa ranked by logistic regression coefficient magnitude. Bars represent log-odds coefficients from the final model. Red bars indicate taxa positively associated with the High Glucose group (positive coefficients); blue bars indicate taxa inversely (negatively) associated with the High Glucose group (negative coefficients). The magnitude of the *F. nucleatum* subsp. *vincentii* coefficient substantially exceeds all others.

All 37 bacterial features that survived the correlation filtering step received non-zero coefficients in the L1-regularized model, indicating that each contributed independently to glucose classification after accounting for multicollinearity. The top 10 most influential taxa, ranked by absolute coefficient value, are summarized in Table 3, and the top 20 are depicted in Figure 6.

**Table 3:** Top 10 most influential bacterial taxa in the L1-regularized logistic regression model.

Bacterial taxon	Coefficient	Direction	*p*-value
*Fusobacterium nucleatum* subsp. *vincentii*	+3.624	Positively associated	< 0.001
*Capnocytophaga sputigena*	+0.550	Positively associated	< 0.001
*Streptococcus intermedius*	+0.517	Positively associated	ns
*Streptococcus sanguinis*	+0.502	Positively associated	< 0.001
*Treponema socranskii*	+0.494	Positively associated	< 0.001
*Campylobacter rectus*	+0.464	Positively associated	< 0.001
*Eikenella corrodens*	+0.451	Positively associated	< 0.001
*Aggregatibacter actinomycetemcomitans*	−0.882	Inversely associated	< 0.001
*Capnocytophaga ochracea*	−0.414	Inversely associated	< 0.001
*Fusobacterium nucleatum* subsp. *polymorphum*	−0.401	Inversely associated	< 0.001

Coefficients are log-odds units from the final model fit on all 8,173 samples with C = 1.0. Positive coefficients indicate a higher predicted probability of High Glucose group membership (a positive association); negative coefficients indicate an inverse (negative) association. Coefficient signs denote direction of association only and do not imply causation. *p*-values are from Mann–Whitney U tests comparing relative abundances between groups. ns, not statistically significant at *p* < 0.05.

The taxon most strongly positively associated with the High Glucose group was *Fusobacterium nucleatum* subsp. *vincentii* (coefficient = +3.624), with a magnitude approximately six times greater than the next highest coefficient. Other taxa positively associated with the High Glucose group, with coefficients above 0.45, included *Capnocytophaga sputigena* (+0.550), *Streptococcus intermedius* (+0.517), *Streptococcus sanguinis* (+0.502), *Treponema socranskii* (+0.494), *Campylobacter rectus* (+0.464), and *Eikenella corrodens* (+0.451). The taxon most strongly inversely (negatively) associated with the High Glucose group was *Aggregatibacter actinomycetemcomitans* (coefficient = −0.882), followed by *Capnocytophaga ochracea* (−0.414) and *Fusobacterium nucleatum* subsp. *polymorphum* (−0.401).

### Abundance comparison of top predictive bacteria

**Figure 7:**
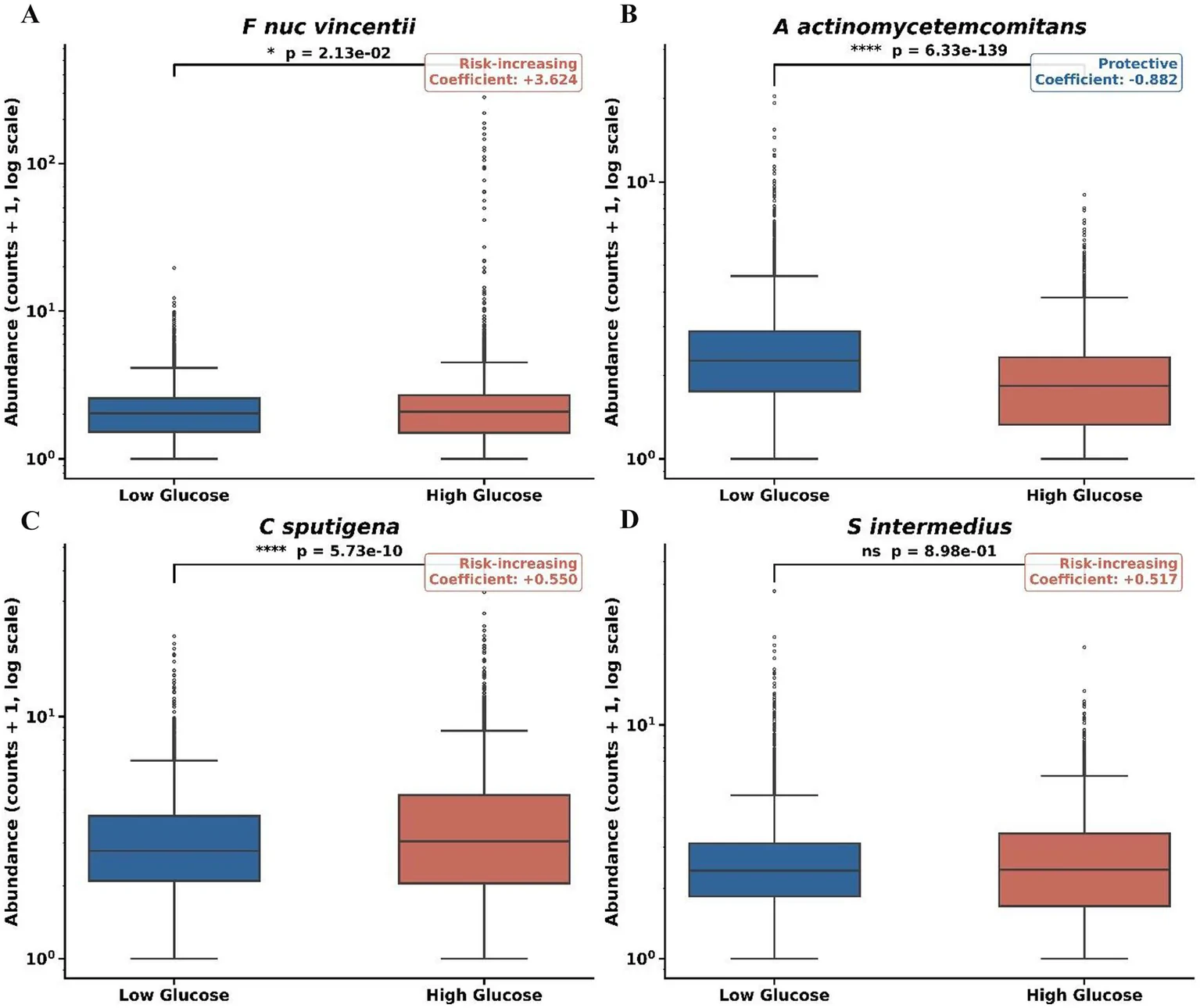
Relative abundance of the four most influential bacterial taxa in low glucose (≤ median) and high glucose (> median) groups. Box plots display the median, interquartile range, whiskers (1.5 × IQR), and individual outliers. Abundances are displayed on a log scale to accommodate the right-skewed, zero-inflated distributions characteristic of microbiome data. **(A)** *F. nucleatum* subsp. *vincentii* (positively associated, coefficient = +3.624); **(B)** *A. actinomycetemcomitans* (inversely associated, coefficient = −0.882); **(C)** *C. sputigena* (positively associated, coefficient = +0.550); **(D)** *S. intermedius* (positively associated, coefficient = +0.517). *p < 0.05; ***p < 0.001; ns, not significant; Mann–Whitney U test.

Box plot analysis of the four most influential bacterial taxa confirmed that the directionality of model coefficients was supported by measurable differences in relative abundance between glucose groups (Figure 7). Given the right-skewed, zero-inflated distributions characteristic of microbiome relative abundance data, abundances are displayed on a log scale to facilitate visualization of group differences across the full dynamic range. *Fusobacterium nucleatum* subsp. *vincentii* showed markedly higher relative abundance in the High Glucose group (*p* < 0.021), with a substantial proportion of high-abundance observations in the high-glucose samples. Consistent with its inverse (negative) coefficient, *Aggregatibacter actinomycetemcomitans was notably more abundant in the Low Glucose group (p < 0.001). Capnocytophaga sputigena was notably more abundant in the High Glucose* group (*p* < 0.001). *Streptococcus intermedius* showed a trend toward higher abundance in the High Glucose group that did not reach statistical significance in the Mann–Whitney U test (*p* = ns), despite its relatively strong model coefficient, suggesting that its predictive contribution may arise from complex covariate relationships captured by the regularized model rather than a simple shift in marginal abundance.

## Discussion

This study demonstrates that the composition of the oral microbiome reflects salivary glucose status in a manner that a regularized machine learning classifier can meaningfully detect. In a cohort of 8,173 Kuwaiti adolescents, participants with higher salivary glucose exhibited lower oral microbial diversity and a recognizably distinct bacterial profile. The model identified individuals with elevated salivary glucose with a sensitivity of 73.9% and an overall accuracy of 75.3%. These results are particularly notable because elevated salivary glucose in this cohort is not a proxy for diagnosed diabetes; rather, it reflects a subclinical metabolic perturbation that can be present in children who are otherwise of normal weight and normotensive (Goodson et al., 2017; Goodson et al., 2014). Prior work has demonstrated that salivary glucose rises in concert with blood glucose and can signal early metabolic dysregulation before any clinical threshold is crossed (Mascarenhas et al., 2014; Hartman et al., 2015; Goodson et al., 2017).

Several groups have characterized the oral microbiome in adult patients with metabolic disorders and reported consistent patterns of dysbiosis. Culture-independent studies using 16S rRNA amplicon sequencing have documented decreased alpha diversity, enrichment of gram-negative anaerobes including *Fusobacterium*, and depletion of commensal taxa in the saliva of individuals with elevated glucose relative to normoglycemic controls (Saeb et al., 2019; Matsha et al., 2020; Gao et al., 2022). Machine learning classifiers applied to salivary microbiome data in adult cohorts with confirmed T2D have achieved discriminatory accuracy values ranging from approximately 0.80 to 0.92, though those findings were derived from clinical samples rather than population-based samples of healthy children (Liu et al., 2021; Gao et al., 2022). The ROC AUC of 0.820 achieved in the present study is comparable to the performance of the salivary microbiome diagnostic model for T2D in Chinese adults reported by Liu et al. (2021) (reported AUC ≈ 0.80), and falls below the cross-cohort meta-analytic model by Gao et al. (2022), which achieved AUC = 0.92 using 470 pooled samples from five studies. The differences in performance are expected, given that the present study targets a pre-clinical pediatric population defined by the cohort median rather than by clinical diagnosis, an inherently more subtle discrimination task than separating confirmed diabetics from matched healthy controls. It is worth noting that the microbiome signal did not depend on how we defined the outcome. The same bacterial features separated the Low and High Glucose groups in the binary model (ROC AUC = 0.820), yet showed almost no linear correlation when glucose was treated as continuous (Spearman *ρ* = −0.005). These two results are consistent rather than at odds: they suggest the microbiome reflects a broad split between higher and lower glucose rather than small continuous shifts, and that the signal holds up whichever way the outcome is analyzed. Our modeling choices were made with this difficulty, and with the nature of the data, in mind. Microbiome relative abundances are compositional, high-dimensional, and strongly correlated, which can destabilize an unpenalized logistic regression and inflate coefficient variance. We therefore used L1 (Lasso) regularization, which adds a penalty proportional to the sum of the absolute coefficients and drives the coefficients of uninformative and redundant taxa to exactly zero (Tibshirani, 1996). This yields a sparse, interpretable model in which the retained taxa can be read directly as the discriminating features, an approach that is now standard in microbiome classification (Knights et al., 2011; Ditzler et al., 2015; Pasolli et al., 2016; Marcos-Zambrano et al., 2021). We deliberately favored this transparent linear model over higher-capacity alternatives such as random forests or gradient boosting; although such methods can sometimes extract additional signal, they are harder to interpret biologically and more prone to overfitting at this feature-to-sample ratio, and the interpretability of the coefficients was central to our aim of identifying candidate taxa.

Saeb et al. (2019) characterized the oral microbiome of T2D and pre-diabetes patients using 16S sequencing and found reduced diversity across both disease states relative to normoglycemic controls, with the pre-diabetes group showing an intermediate diversity profile. The findings of the present study align with this gradient: the median-based dichotomization captures early, subclinical glucose elevation rather than frank hyperglycemia, and the diversity reduction observed is modest in absolute terms (Shannon: 3.229 vs. 3.188) but highly statistically significant at this cohort size, consistent with an early-stage signal.

The glucose microbiome relationship observed in this cohort is biologically plausible through several converging mechanisms. At the local level, elevated salivary glucose provides a fermentable substrate that strongly favors acidogenic Firmicutes, which constitute approximately 39.9–44.2% of salivary bacteria and are capable of rapidly converting glucose to organic acids (Goodson et al., 2017; Cena et al., 2023). This mechanism is consistent with the threshold kinetics of salivary glucose described in pediatric cohorts, in which glucose accumulates in saliva only once blood glucose exceeds the reabsorptive capacity of the ductal transporters, at which point the newly available substrate is fermented to acid and shifts the local ecology toward acidogenic and acid-tolerant taxa (Hartman et al., 2015; Goodson et al., 2017). Because this substrate-driven selection operates continuously rather than at a single clinical cut-point, even the modest, subclinical elevation captured by our median split would be expected to produce a measurable, directional change in community composition, which is what we observe. This acidification selectively suppresses acid-sensitive commensal species, effectively narrowing the microbial community and generating the diversity reduction observed in the High Glucose group. At the systemic level, the pro-inflammatory products of dysbiotic oral communities, including lipopolysaccharide from gram-negative anaerobes and proteases from *Porphyromonas gingivalis* and *Fusobacterium*, gain access to the circulation through the highly vascularized periodontal tissues and inflamed gingival sulcus (Hajishengallis, 2015; Liccardo et al., 2019). These molecules activate systemic toll-like receptor pathways, stimulate production of tumor necrosis factor-alpha and interleukin-6, and impair insulin receptor substrate phosphorylation, thereby reducing peripheral glucose uptake (Xiao et al., 2017; Graves et al., 2019). The IL-17 signaling axis plays a central role in this process: diabetes-driven IL-17 overexpression reshapes subgingival communities toward greater pathogenicity, a change that can be partially reversed by IL-17 inhibition (Moskovitz et al., 2021). In the developing immune systems of adolescents, these effects may be particularly consequential because the inflammatory set points established during puberty can track into adulthood.

The finding that Shannon and Simpson diversity indices are notably lower in the High Glucose group resonates with a well-established pattern in the gut microbiome literature, where reduced diversity has been consistently reported in individuals with T2D, impaired fasting glucose, and obesity (Saeb et al., 2019; Yuan et al., 2022). In gut microbiome studies, decreased diversity typically reflects the loss of protective commensal species, including butyrate producers, with downstream consequences for intestinal permeability and systemic inflammation (Yuan et al., 2022). In the oral cavity, the ecological interpretation is somewhat different: the oral ecosystem maintains a tightly regulated balance between acidogenic, aciduric, and commensal species, and perturbation of this balance in either direction can have consequences for both local and distant tissues (Negrini et al., 2021). There are at least two mechanistic explanations for the observed diversity reduction. First, elevated salivary glucose provides a fermentable substrate that selectively advantages acidogenic species, effectively narrowing the microbial community (Goodson et al., 2017; Cena et al., 2023). Second, the systemic inflammatory state associated with impaired glucose regulation promotes heightened IL-17 signaling in the periodontium, which shifts the subgingival environment toward a dysbiotic state characterized by enrichment of gram-negative anaerobes (Moskovitz et al., 2021). Whether the diversity reduction precedes or follows the glucose elevation cannot be resolved from the cross-sectional design of this study, but mechanistic animal models suggest that hyperglycemia actively drives microbial pathogenicity rather than merely reflecting it (Xiao et al., 2017; Graves et al., 2019).

The extraordinary predictive weight assigned to *F. nucleatum* subsp. *vincentii* (coefficient = +3.624, approximately six times the magnitude of the next highest coefficient) is the most striking finding of this study and warrants careful interpretation. *Fusobacterium nucleatum* is a gram-negative, obligate anaerobe that occupies a keystone position in dental plaque biofilm architecture, serving as a coaggregation bridge between early and late colonizers (Fan et al., 2022). Its systemic reach has been documented in associations with colorectal cancer, adverse pregnancy outcomes, inflammatory bowel disease, cardiovascular disease, and rheumatoid arthritis (Fan et al., 2022). In the context of glucose metabolism, the relevant biology centers on its capacity to produce pro-inflammatory lipopolysaccharide and short-chain fatty acids that enter the systemic circulation, activate toll-like receptor signaling, and impair insulin receptor phosphorylation (Hajishengallis, 2015; Liccardo et al., 2019).

The subspecies distinction is biologically important and directly supported by the present data. In our model, *F. nucleatum* subsp. *vincentii* received the highest positive coefficient of all 37 retained bacterial features (coefficient = +3.624), while *F. nucleatum* subsp. *polymorphum* received a negative coefficient (−0.401), indicating an inverse (negative) rather than positive association with salivary glucose elevation. This divergence within what has historically been treated as a single species is biologically meaningful. The four recognized subspecies of *F. nucleatum* (*nucleatum*, *vincentii*, *polymorphum*, and *animalis*) differ substantially in their adhesin profiles, virulence factor expression, and clinical disease associations (Fan et al., 2022). Recent work has demonstrated that these subspecies are not interchangeable in their pathogenic potential and that their disease associations depend heavily on the local microbial and clinical context (Krieger et al., 2024a, 2024b). The opposing directions of the two *F. nucleatum* subspecies in our model align with this picture and may help explain some of the inconsistency in the prior *Fusobacterium*–metabolic disease literature. These findings highlight the importance of resolving microbiome data to the subspecies level in future studies of oral bacteria and systemic health. Comparative genomic work supports the biological basis for this divergence: a pangenomic analysis of *F. nucleatum* found that each subspecies carries a distinct set of accessory gene clusters, and that virulence genes, antibiotic-resistance genes, and genomic islands are distributed unevenly across subspecies and even across strains within a subspecies (Ma et al., 2023). The same work, together with recent clinical surveys, notes that subsp. *vincentii* and subsp. *polymorphum* occupy different ecological niches and differ in their disease associations despite their close relatedness (Ma et al., 2023; Krieger et al., 2024a). The opposing coefficients we observe for these two subspecies are therefore consistent with genuine functional differences rather than measurement noise, and they reinforce the argument that collapsing *F. nucleatum* to the species level can obscure clinically meaningful signal.

The negative coefficient for *A. actinomycetemcomitans* (−0.882) is counterintuitive at first glance, given that this bacterium is historically classified as a principal etiological agent of aggressive periodontitis. However, inverse (negative) association in this cohort is consistent with recent observations that the ecological context in which a pathogen is found matters as much as the pathogen’s intrinsic virulence. Matsha et al. (2020) similarly observed complex and sometimes counterintuitive associations between known periodontal pathogens and glycemic status in their South African cohort, particularly when the degree of glycemic control was taken into account. An alternative interpretation is ecological exclusion: *A. actinomycetemcomitans* competes with *F. nucleatum* for niches within the oral biofilm, and its higher abundance in the Low Glucose group may partly reflect competitive suppression of the fusobacterial populations associated with the high-glucose phenotype.

The positively associated taxa ranked below *F. nucleatum* subsp. *vincentii* encompass a phylogenetically diverse set of organisms with established roles in periodontal pathogenesis. *Capnocytophaga sputigena is a gram-negative gliding rod of the green complex, while C. rectus* (*Campylobacter rectus*) is a motile orange-complex rod; both are associated with periodontitis and systemic inflammation, and their elevated abundance in high-glucose samples is consistent with a dysbiotic shift toward periodontal pathogens (Hajishengallis, 2015). *Treponema socranskii* is a spirochete associated with advanced periodontal disease, and its enrichment further supports the hypothesis that the high-glucose microbiome resembles the community structure observed in periodontally compromised adults (Matsha et al., 2020). *Streptococcus sanguinis* and *S. intermedius* are gram-positive commensals that are also opportunistic pathogens; their higher abundance in the high-glucose group may reflect the selective advantage that carbohydrate-fermenting species gain in a glucose-enriched salivary environment (Negrini et al., 2021).

The oral microbiome of children with type 1 diabetes has been characterized by Moskovitz et al. (2021) in a cohort of 37 diabetic and 29 healthy children aged 5–15 years, using 16S rRNA gene sequencing, which identified elevated *Streptococcus* abundance and taxa associated with gut microbiome changes in type 1 diabetes. Janem et al. (2017) examined obese children with and without T2D (*n* = 49 total) and found elevated salivary inflammatory markers and a distinct microbiome in the diabetic subgroup. The present study extends these findings, demonstrating that distinct oral microbiome compositions are detectable in children with elevated salivary glucose even in the absence of a formal metabolic disease diagnosis.

The oral microbiome classifier in this study achieved a ROC AUC of 0.820, which is comparable to the discriminatory performance of fasting blood glucose testing (approximately 0.75) and approaches the combined performance of fasting blood glucose plus HbA1c (approximately 0.84) for detecting impaired glucose tolerance in population screening (Mascarenhas et al., 2014). This comparison is indirect: the cited AUCs for fasting blood glucose and HbA1c derive from different populations and outcome definitions, and no head-to-head comparison against these established biomarkers was performed in the present study. The numbers are therefore offered only as a rough frame of reference and not as evidence of equivalent clinical performance. The practical significance of this finding lies in the non-invasive nature of saliva collection, which may make it worth evaluating as a non-invasive approach for large-scale metabolic screening in school or community settings, pending external validation.

### Limitations

Several limitations of this study must be acknowledged. First, the cross-sectional design precludes causal inference: it is not possible to determine from these data whether the observed microbiome shifts drive glucose elevation, arise as a consequence of it, or are mediated by a shared upstream factor such as diet. Longitudinal studies tracking both oral microbiome composition and glycemic trajectories are needed to address this question (Sanz et al., 2018). Although age, sex, and BMI were addressed directly through the supplementary confounder analysis described in the Results, other variables that were not recorded in the Kuwait Healthy Life Study, in particular diet, oral hygiene practices, and socioeconomic status, could not be adjusted for, and these may influence both salivary glucose and microbiome composition. Second, the DNA probe method, while well-validated for the 42 targeted species used in the Kuwait Healthy Life Study, captures a pre-specified panel rather than the full oral metagenome. The checkerboard platform measures a fixed, validated set of probes rather than surveying the full community of 700 or more oral taxa (Socransky and Haffajee, 2005; Baker et al., 2024). Species not included in the probe set may carry additional predictive information, and functional profiling through shotgun metagenomics and metabolomics could reveal metabolic pathway alterations that are invisible to species-level compositional data. Because the checkerboard assay is hybridization-based rather than sequence-based, it cannot characterize gene content or metabolic pathways, and it cannot detect novel or uncultured taxa that fall outside the 42-species panel (Goodson et al., 2017); the full list of species measured is given in Supplementary Table S1. Future work on this cohort would benefit from 16S rRNA amplicon sequencing for finer taxonomic resolution and from shotgun metagenomics paired with metabolomics to address functional questions directly. Third, the median dichotomization of salivary glucose creates two groups that differ by only a narrow margin near the cutoff. Future studies using quantile-based or clinically defined thresholds, or treating glucose as a continuous outcome in a regression framework, would complement the classification approach taken here. Fourth, the cohort is drawn from a single country (Kuwait) and a narrow age range (approximately 9–11 years), limiting geographic and age generalizability. The microbiome composition of Kuwaiti children reflects specific dietary, cultural, and genetic factors that may differ substantially from other populations; validation in independent cohorts from different ethnic and geographic backgrounds is essential before clinical translation (Baker et al., 2024). Fifth, while the logistic regression model is interpretable and well-suited to the available sample size, more complex approaches such as random forests, gradient boosting, or neural networks might extract additional predictive signal, particularly if functional metagenomic features were incorporated.

## Conclusion

This study provides evidence that the oral microbiome of children carries a detectable signature of their salivary glucose status. In a cohort of 8,173 Kuwaiti adolescents, those with higher salivary glucose exhibited a less diverse oral microbial community and a distinct bacterial composition linked to metabolic and inflammatory dysregulation. A classification model trained on bacterial species correctly identified children with elevated salivary glucose at a level of discriminatory accuracy comparable to conventional blood-based metabolic tests, using a non-invasive saliva sample. Importantly, none of the children in this cohort carried a formal diagnosis of diabetes or metabolic disease, yet the oral microbiome still reflected differences in glucose metabolism that the model could detect. This suggests that the oral cavity may begin to manifest signs of metabolic imbalance at a subclinical stage, before clinical thresholds of dysglycemia are crossed, precisely the window in which early intervention could have the greatest impact on long-term metabolic trajectories.

The oral microbiome is a personalized biological fingerprint that reflects the host’s immune and genetic profile and is responsive to systemic metabolic perturbations. The identification of specific bacterial taxa, most prominently *F. nucleatum* subsp. v*incentii*, as strong predictors of elevated salivary glucose provides mechanistic targets for future investigation and potential microbiome-directed interventions. Translation of these findings into clinical practice will require longitudinal validation and replication in independent, ethnically diverse cohorts. Nonetheless, the results presented here support the concept that a simple, non-invasive oral sample analyzed for bacterial composition could serve as a candidate non-invasive marker warranting prospective evaluation for children. Whether such profiling can identify individuals at risk in advance of clinical manifestation of dysglycemia is a hypothesis that will require longitudinal validation.

## Data Availability

Publicly available datasets were analyzed in this study. This data can be found at: https://doi.org/10.1371/journal.pone.0170437.s001.
