# Supplementary material for: Oral microbiome classification of elevated salivary glucose in a large adolescent Kuwaiti population
Source: Front Microbiol. 2026 Aug 12;17:1863859. doi: 10.3389/fmicb.2026.1863859 (PMC13510292; doi:10.3389/fmicb.2026.1863859)
Supplement: Supplementary file 1 [file Supplementary_file_1.docx]

# Supplementary Material

*Oral microbiome classification of elevated salivary glucose in a large adolescent Kuwaiti population*

Desai C, Alsediqi A, Alsanea A, Albader F, Jomah H, Alkandari H, Alqaderi H

## Supplementary Table S1. The 42-species checkerboard panel, grouped by Socransky complex

The Kuwait Healthy Life Study profiled saliva using the whole-genomic DNA probe (Socransky checkerboard) method, which measures a fixed, pre-specified panel of 42 bacterial species (Goodson et al., 2017). The species are listed below, organized by the microbial complexes described by Socransky and colleagues (Socransky et al., 1998), with the blue (Actinomyces) complex added in later work (Socransky and Haffajee, 2002). Species not assigned to one of the original 1998 complexes, including the caries-associated and commensal taxa on the saliva panel, are grouped under “Other.”

| **Bacterial species** | **Socransky complex** | **Notes** |
| --- | --- | --- |
| *Porphyromonas gingivalis* | Red | Keystone periodontal pathogen |
| *Tannerella forsythia* | Red | Formerly Bacteroides forsythus |
| *Treponema denticola* | Red | Oral spirochete |
| *Fusobacterium nucleatum subsp. nucleatum* | Orange | Biofilm bridging species |
| *Fusobacterium nucleatum subsp. vincentii* | Orange | Most strongly positively associated taxon in this study |
| *Fusobacterium nucleatum subsp. polymorphum* | Orange | Inverse (negative) association in this study |
| *Fusobacterium periodonticum* | Orange | Orange-complex anaerobe |
| *Prevotella intermedia* | Orange | Gram-negative anaerobe |
| *Prevotella nigrescens* | Orange | Gram-negative anaerobe |
| *Parvimonas micra* | Orange | Formerly Peptostreptococcus micros |
| *Campylobacter rectus* | Orange | Motile gram-negative rod |
| *Campylobacter showae* | Orange | Motile gram-negative rod |
| *Campylobacter gracilis* | Orange | Motile gram-negative rod |
| *Eubacterium nodatum* | Orange | Gram-positive anaerobe |
| *Streptococcus constellatus* | Orange | Anginosus group streptococcus |
| *Streptococcus sanguinis* | Yellow | Early colonizer; formerly S. sanguis |
| *Streptococcus oralis* | Yellow | Commensal early colonizer |
| *Streptococcus mitis* | Yellow | Commensal early colonizer |
| *Streptococcus gordonii* | Yellow | Commensal early colonizer |
| *Streptococcus intermedius* | Yellow | Anginosus group; opportunistic |
| *Aggregatibacter actinomycetemcomitans* | Green | Most strongly inversely associated taxon in this study |
| *Capnocytophaga gingivalis* | Green | Gram-negative facultative |
| *Capnocytophaga sputigena* | Green | Positively associated taxon in this study |
| *Capnocytophaga ochracea* | Green | Inverse (negative) association in this study |
| *Campylobacter concisus* | Green | Gram-negative rod |
| *Eikenella corrodens* | Green | Facultative gram-negative rod |
| *Veillonella parvula* | Purple | Lactate-utilizing anaerobe |
| *Actinomyces odontolyticus* | Purple | Early colonizer |
| *Actinomyces naeslundii* | Blue | Early colonizer |
| *Actinomyces viscosus* | Blue | Early colonizer |
| *Streptococcus mutans* | Other | Principal cariogenic species |
| *Streptococcus salivarius* | Other | Abundant salivary commensal |
| *Streptococcus anginosus* | Other | Anginosus group streptococcus |
| *Cutibacterium acnes* | Other | Formerly Propionibacterium acnes |
| *Neisseria mucosa* | Other | Abundant salivary commensal |
| *Prevotella melaninogenica* | Other | Abundant salivary anaerobe |
| *Selenomonas noxia* | Other | Motile anaerobe |
| *Leptotrichia buccalis* | Other | Gram-negative anaerobe |
| *Gemella morbillorum* | Other | Gram-positive commensal |
| *Actinomyces israelii* | Other | Gram-positive filament |
| *Treponema socranskii* | Other | Oral spirochete; positively associated in this study |
| *Propionibacterium acidifaciens* | Other | Acidogenic anaerobe |

*Complex groupings follow Socransky et al. (1998): the red and orange complexes are most strongly associated with periodontal disease, whereas the yellow, green, purple, and blue complexes are generally compatible with periodontal health.*

## Supplementary Table S2. Logistic regression coefficients for the top 20 predictive taxa

Coefficients are log-odds units from the L1-regularized logistic regression model fitted on the microbiome features alone (the primary model), ranked by absolute magnitude. Positive coefficients indicate a higher predicted probability of High Glucose group membership (a positive association); negative coefficients indicate an inverse (negative) association. Coefficient signs denote direction of association only and do not imply causation.

| **Rank** | **Bacterial species** | **L1 coefficient** | **Direction** |
| --- | --- | --- | --- |
| 1 | *Fusobacterium nucleatum subsp. vincentii* | +3.624 | Positively associated |
| 2 | *Aggregatibacter actinomycetemcomitans* | −0.882 | Inversely associated |
| 3 | *Capnocytophaga sputigena* | +0.550 | Positively associated |
| 4 | *Streptococcus intermedius* | +0.517 | Positively associated |
| 5 | *Streptococcus sanguinis* | +0.502 | Positively associated |
| 6 | *Treponema socranskii* | +0.494 | Positively associated |
| 7 | *Campylobacter rectus* | +0.464 | Positively associated |
| 8 | *Eikenella corrodens* | +0.451 | Positively associated |
| 9 | *Capnocytophaga ochracea* | −0.414 | Inversely associated |
| 10 | *Fusobacterium nucleatum subsp. polymorphum* | −0.401 | Inversely associated |
| 11 | *Cutibacterium acnes* | −0.333 | Inversely associated |
| 12 | *Treponema denticola* | −0.302 | Inversely associated |
| 13 | *Capnocytophaga showae* | +0.288 | Positively associated |
| 14 | *Streptococcus mutans* | −0.281 | Inversely associated |
| 15 | *Streptococcus salivarius* | −0.276 | Inversely associated |
| 16 | *Actinomyces viscosus* | +0.271 | Positively associated |
| 17 | *Porphyromonas gingivalis* | −0.260 | Inversely associated |
| 18 | *Streptococcus oralis* | −0.229 | Inversely associated |
| 19 | *Actinomyces naeslundii* | −0.225 | Inversely associated |
| 20 | *Streptococcus anginosus* | −0.192 | Inversely associated |

*This table reports coefficients from the microbiome-only model (Model A). The coefficient ranking and direction from the confounder-adjusted model (Model C, which additionally includes age, sex, and BMI) were consistent with those shown here.*

## Supplementary references

Goodson, J. M., Hartman, M. L., Shi, P., Hasturk, H., Yaskell, T., Vargas, J., et al. (2017). The salivary microbiome is altered in the presence of a high salivary glucose concentration. PLoS One 12:e0170437. doi: 10.1371/journal.pone.0170437

Socransky, S. S., Haffajee, A. D., Cugini, M. A., Smith, C., and Kent, R. L. Jr. (1998). Microbial complexes in subgingival plaque. J. Clin. Periodontol. 25, 134–144. doi: 10.1111/j.1600-051X.1998.tb02419.x

Socransky, S. S., and Haffajee, A. D. (2002). Dental biofilms: difficult therapeutic targets. Periodontol. 2000 28, 12–55. doi: 10.1034/j.1600-0757.2002.280102.x
